# 

**Published:** 1899-07-15

**Authors:** 


					THE HOSPITAL. ?< The standard of highest purity."?Lancet, July 15th, 1899.
CADBURY'S j** ijr fe* CADBURY'S
COCOA % MM COCOA
ABSOLUTELY PURR. NO DRUGS OR ALKALIES USED.
Lflscofr Western Infirmary
No. M8j Vol. XXVI. GK^P6?]
Saturday, July 15th, 1899.
- AJa NbfitYfCH Hfi*o/TAL
[Sabsc^ptonTenHB.] pRICE TWOpENCE.

				

